# One Institution’s evaluation of family medicine residency applicant data for academic predictors of success

**DOI:** 10.1186/s12909-021-02518-w

**Published:** 2021-02-02

**Authors:** Michael E. Busha, Brock McMillen, Jeffrey Greene, Kristine Gibson, Charlotte Milnes, Peter Ziemkowski

**Affiliations:** 1 Western Michigan University Homer Stryker M.D. School of Medicine, 1000 Oakland Drive, Kalamazoo, MI 49008 USA; 2 Indiana University School of Medicine, 1520 North Senate, Indianapolis, IN 46202 USA

**Keywords:** Family medicine, Resident recruitment, Predictive value, Academic record

## Abstract

**Background:**

Family Medicine residencies are navigating recruitment in a changing environment. The consolidation of accreditation for allopathic and osteopathic programs, the high volume of applicants, and the forthcoming transition of the United States Medical Licensing Exam (USMLE) Step 1 to pass/fail reporting all contribute. This retrospective cohort study evaluated which components of a student’s academic history best predict readiness for residency.

**Methods:**

In 2020, we analyzed applicant data and initial residency data for program graduates at a single residency program between 2013 and 2020. This included undergraduate education characteristics, medical school academic performance, medical school academic problems (including professionalism), STEP exams, location of medical school, and assessments during the first 6 months of residency. Of 110 matriculating residents, assessment data was available for 97 (88%).

**Results:**

Pre-matriculation USMLE data had a positive correlation with initial American Board of Family Medicine (ABFM) in-training exams. Pre-matriculation exam data did not have a positive correlation with resident assessment across any of the six Accreditation Council for Graduate Medical Education (ACGME) competency domains. A defined cohort of residents with a history of academic struggles during medical school or failure on a USMLE exam performed statistically similarly to residents with no such history on assessments across the six ACGME competency domains.

**Conclusions:**

Applicants with a history of academic problems perform similarly in the clinical environment to those without. While a positive correlation between pre-matriculation exams and the ABFM in-training exam was found, this did not extend to clinical assessments across the ACGME competency domains.

## Background

According to the National Resident Match Program (NRMP), Family Medicine offered 4662 positions in the 2020 NRMP Match, which was 555 more than 2019 and representative of 13.7% of all positions across disciplines [1]. In order to fill these positions, Family Medicine residency programs must allocate a significant amount of time and resources to narrowing their applicant pool; the residency in which this study occurs has thirteen positions in the NRMP each year and routinely exceeds 100 applicants per position. Distinguishing applicants that are both prepared for the academic rigor of residency and a good fit for the residency culture is important to program success.

The transformation of undergraduate medical education (UME) from a solely time-based education curriculum with traditional grading to a competency-based assessment system has complicated this recruitment process. In place of a traditional grading system, undergraduate medical students are being evaluated in terms of developmental growth across competencies. This creates a broader assessment framework than traditional grading on content mastery through exams. The Association of American Medical Colleges (AAMC) recognizes eight competency domains which are commonly used by medical schools during design of their assessment framework. They include patient care, knowledge for practice, practice-based learning and improvement, interpersonal and communication skills, professionalism, systems-based practice, interprofessional collaboration, and personal and professional development. Furthermore, in line with UME curriculum changes, the National Board of Medical Examiners (NBME) has announced an intention to change the score reporting on USMLE Step 1 to pass/fail on or after January 2022 [2]. Historically, USMLE Step 1 scores have been one of the critical pieces of data residency directors across most specialties have used to screen applicants [3, 4]. The loss of a scaled score will significantly impact the initial applicant screening process and also increase the stakes of an initial USMLE failing exam by applicants. Reviewers will no longer be able to contextualize a failing score in the same way with other application materials and a subsequent scaled score demonstrating degree of improvement.

As many interview components transition to the virtual realm, medical student away rotations decline, and dynamics in student data continue to change, Family Medicine Residencies face significant new recruitment challenges and must determine how best to screen and evaluate applicants. The COVID-19 pandemic has hastened many of these trends for the NRMP 2021 match season creating further uncertainty on future recruitment practices.

While prior residency studies across a spectrum of specialties have delineated criteria used to select students for interviews [4] as well as criteria for ranking resident candidates for acceptance into their program [5–7], few have been able to find valid or effective proxy measures for predicting resident success [3, 8, 9]. The literature in the discipline of Emergency Medicine supports the most predictive measure of future performance in residency as the Rank Order List placement for those students who had rotated as a sub-intern in their residency program during their fourth-year of medical school. Despite the high rank USMLE Step 1 scores held in early calculations to grant an interview or where to rank a student, they did not predict performance on patient care and professional competencies [7]. Predictive tools for assessing applicant academic history remain mixed across specialties. Unfortunately, Family Medicine specific literature on academic predictors of residency performance is lacking.

In this study, we reviewed data from a single, academic-health-center based Family Medicine residency program. The purpose of this study was to first determine whether a positive correlation exists between USMLE results, American Board of Family Medicine (ABFM) in-training exam results, and clinical competency assessments in Family Medicine and compare any differences with reported literature findings across different specialties. We then studied selected applicant characteristics (type and location of medical school attended, length of time between undergraduate degree conferment and medical school matriculation and a history of academic struggles during medical school) to determine predictive value in medical student readiness for residency. Our goal was to establish correlation data that could assist program directors in better evaluating these components of an applicant’s academic history. We were specifically seeking direct predictors of higher clinical performance compared to cohort, predictors of lower clinical performance compared to cohort, and identifying commonly used ‘red flags’ to determine if significance existed.

## Methods

The Indiana University-Methodist Family Medicine Residency program is based in an urban academic health center. Residency recruitment occurs through the NRMP with a range of 10–15 positions each match year during the study period. Subjects of the study include all matriculating residents for the graduating cohorts from June 2013 through June 2020, which includes 8 years of matriculating cohort data. Research occurred in 2020.

Data collection included extraction from Electronic Residency Application Service (ERAS) applications, Medical Student Performance Evaluations (MSPE) formerly known as the Dean’s Letter, USMLE transcripts, ABFM in-training exam results and a composite of resident clinical assessments on the family medicine inpatient service during the first 6 months of training across the ACGME competency domains.

The ABFM in-training exam is a nationally benchmarked exam assessing the general content areas within the scope of Family Medicine. It mirrors in content and scoring methodology the ABFM specialty certification exam. The Family Medicine inpatient competency assessment was created utilizing the developmental behaviors associated with milestones across the ACGME competency domains as defined by the ACGME Family Medicine Residency Review Committee. The faculty group determined which milestones were appropriate for an inpatient environment including areas across all six domains. The tool was 25 questions in length. Length, content and value of data obtained were evaluated by the faculty committee and a subset of the Clinical Competency Committee through a three-month pilot prior to finalization and implementation (including comparisons with prior assessment form). Assessments were assigned to service faculty at the conclusion of each service week with an assigned resident. Composite assessment reports were generated allowing the utilization of quantitative data with a range of three to eight evaluators. The assessment scale utilized mirrored the ACGME Family Medicine milestones with one representing a novice resident, two representing an advanced beginner resident, three representing a competent resident, four representing proficiency (graduation target) and five representing an attending or expert resident performance in the individual milestone being assessed.

All matriculating residents through the NRMP were included in this analysis, including those who eventually transferred into another residency/specialty or separated from the residency due to personal or academic reasons (5). There were also three students who entered the program outside of the NRMP, off-cycle from the July 1 traditional start date; those residents did not have ERAS applications to utilize but available data were included.

Data extracted for analysis included all USMLE STEP 1, STEP 2 CK and STEP 2 CS attempts with scores; the ABFM in-training exam delivered in October of the residents’ first year of residency; a student history of undergraduate medical education remediations and professionalism problems as reported in the MSPE and transcript; the dates of undergraduate degree conferment and medical school matriculation; the permanent address of applicant; medical school location; and a composite evaluation from all participating faculty on the Family Medicine inpatient service for the first 6 months of residency for each resident. Residents completed either 4 or 8 weeks of Family Medicine inpatient service with a range of 3–8 assigned evaluators.

We selected two types of statistical procedures for the study. For characteristics measured at the interval level, we computed Pearson correlations using residents’ clinical and academic performance. Using an expected effect size of .3 and an α error probability of .05, our post hoc calculation using G*Power 3.1 yielded a power of .83 for our group sample size of 92. For characteristics amenable to categorical grouping (e.g., US allopathic medical school graduates vs. international medical school graduates or having an in-state permanent address vs. an out-of-state-address), we performed a Welch two-samples *t*-test for comparing clinical and academic performance scores between the groups. Anticipating a large effect size of .8 and using an α error probability of .05, we calculated a power of .87 for these tests for the group sample sizes available to us.

This study was determined to be exempt by the Indiana University School of Medicine Institutional Review Board. The need for informed consent is waived by Indiana University School of Medicine Institutional Review Board as it is secondary research. All methods were carried out in accordance with relevant guidelines and regulations.

## Results

Of 110 residents who entered the program during the study period, complete assessment information was available for 97 (88%). The availability of personal information on the 97 applicants varied; not all elements of application data were available for every resident. Matriculating resident characteristics are delineated in Table 1. Mean performance on the ABFM in-training-exam and across the ACGME competency domains as assessed by faculty during resident’s Family Medicine inpatient rotations are shown in Table 2.

**Table 1:** Matriculant Characteristics

Female	42%
Months between college graduation and medical school matriculation (average)	20.9
US allopathic medical school graduate	24%
International medical school graduate	73%
Academic (including professionalism) concerns in medical school (%)	19%
Engagement in medical school organizations	34%
Permanent address in-state	20%
Permanent address out-of-state/international	80%
Allopathic medical school located in-state	10%
Allopathic medical school located out-of-state	10%
USMLE Step 1 (average, first-time takers)	209.4
USMLE Step 2 CK (average, first-time takers)	219.6
USMLE Step 2 CS (number failing, first-time takers)	4

**Table 2:** Residency Performance Characteristics

Residency Performance Characteristics	Average	SD
PGY-1 ABFM In-Training-Exam	376.8	70.5
Competency: Patient Care	2.75	.863
Competency: Medical Knowledge	2.69	.845
Competency: Interpersonal Communication Skills	2.73	.944
Competency: Professionalism	2.65	.985
Competency: Composite Average	2.66	.887

Table 3 shows computed correlations among seven academic, clinical, and demographic variables for first-year residents. Results indicated moderate and statistically significant associations greater than or equal to *r*(96) = +.466, *p* < .001 between scores on the PGY1 In-Training Exam and USMLE Step 1 as well as between the PGY1 In-Training Exam and USMLE Step 2 CK. In addition, there were small and statistically significant correlations greater than or equal to *r*(106) = +.213, *p* < .05 between the PGY1 In-Training Exam and the competencies of Patient Care, Medical Knowledge, Professionalism, and the Composite score.

**Table 3:** Descriptive Statistics and Correlation Matrix for Residency Readiness

Variable	n	M	SD	ITE	PC	MK	ICS	Prof	Comp	Gap
Step 1	96	209.4	21.1	.466**	−.003	.008	−.060	−.001	−.051	.036
Step 2 CK	96	219.7	20.9	.475**	−.022	.004	−.103	−.060	−.067	−.218
Gap	72	25.1	29.1	.140	−.148	−.176	−.091	−.067	−.120	–
ITE	106	376.8	70.5	–	.230*	.234*	.190	.224*	.213*	.039

*p < .05

**p < .001

Legend – *ITE* In-Training Exam, *PC* Patient Care, *MK* Medical Knowledge, *ICS* Interpersonal Communication Skills, *Prof* Professionalism, *Comp* Composite Score (average of PC, MK, ICS, and Prof); Gap: Number of months between medical school graduation and beginning of residency

Table 4 displays results from a series of Welch’s *t*-test procedures among several variables and shows that residents with no history of academic problems (including professionalism) scored significantly higher than residents with such a history on the PGY1 In-Training Exam (*t*(49.879) = 2.23, *p* = .030). The remaining comparisons between these two groups did not yield any significant differences. Using the same statistical procedure, analysis of resident readiness variables for students based on permanent address relative to residency location, medical school proximity to residency location and medical school categorization yielded no significant differences between the respective groups.

**Table 4:** Results of Welch’s *t*-test on History of Problems in Academics or Professionalism

Dependent Variable	No Hx of Academic Problems		Hx of Academic Problems		*Welch’s t*(df)	*p*	Cohen’s *d*
	M	SD	M	SD			
ITE	382.5	74.6	353.8	45.7	2.23 (49.879)	0.030*	0.46
PC	2.82	.866	2.52	.830	1.42 (33.053)	.166	0.35
MK	2.79	.825	2.39	.862	1.90 (30.860)	.067	0.47
ICS	2.82	.924	2.40	.962	1.78 (30.955)	.085	0.44
Prof	2.71	.978	2.46	1.00	1.02 (31.256)	.314	0.25
Comp	2.73	.887	2.40	.854	1.56 (33.286)	.127	0.38

*p < .05

Legend – *ITE* In-Training Exam, *PC* Patient Care, *MK* Medical Knowledge, *ICS* Interpersonal Communication Skills, *Prof* Professionalism, *Comp* Composite Score (average of PC, MK, ICS, and Prof)

## Discussion

This study affirms literature from other specialties demonstrating a lack of correlation between USMLE Step 1 and Step 2 scores and clinical performance with a focus on the six ACGME competency domains. Uniquely, this study extends that analysis to academically challenged applicants. The results demonstrate a history of academic challenges in medical school do not correlate to clinical performance across the six ACGME competency domains during the first 6 months of residency. This study suggests using isolated challenges students may have had requiring a remediation or second attempt on a USMLE exam is not statistically warranted in identifying capacity for success in residency.

Further, this study affirms a positive correlation between USMLE Step 1 and Step 2 scores and in-training examinations for incoming residents as established in the literature for other specialties. These findings are consistent with an extensive study involving over 9000 first-year internal medicine residents. The USMLE Step scores were strong predictors of passing the American College of Physicians Internal Medicine In-Training Examination [10]. Another meta-analysis of 80 studies concluded the strongest positive relationship was between USMLE Step 1 and in-training examinations [3]. In Neurology, one study has affirmed the predictive value of USMLE Step 1 scores and future successful performance on standardized medical examinations [11]. Our analysis of residents with a history of academic difficulties also demonstrated a correlation with below cohort mean in-training examination performance.

Using the ACGME core competency domains as a designation of clinical performance, our findings revealed the USMLE Step scores have poor predictive value in Family Medicine. In agreement with our results, an examination of data in obstetrics and gynecology concluded objective data such as the USMLE scores did not correlate with candidates’ overall performance [12]. A Neurology study which affirmed Step 1 predictive value on in-training examination scores also emphasized no correlation to overall resident competence [11]. This is further affirmed by Lee and Vermillion through an examination of 485 graduates of a single institution across multiple specialties where UME data was compared with a standardized internship evaluation sent to program directors; it demonstrated Step 1 was not a strong predictor of internship performance [13]. In a comprehensive systematic review of undergraduate measurements, USMLE scores appear valuable as they relate to success on in-training examinations but are less effective in predicting best clinical performance [9]. Furthermore, McGaghie et al. concluded utilization of USMLE Step 1 and 2 to evaluate applicants is “neither structured, coherent, nor evidence based” [8].

There are some limited studies that have concluded a positive correlation exists between USMLE Step 1 and clinical performance. For example, one contends higher USMLE Step 1 scores correlate with completion of a general surgery residency [14]. Another supports USMLE Step 1 as a positive predictor of resident performance in Emergency Medicine [15].

In consideration of other independent variables, a history of academic problems such as a required remediations during medical school or needing to retake a USMLE exam was examined. Our results demonstrated no predictive value from the existence of academic problems in the application file. This is consistent with Brenner’s examination which found no correlation between a history of academic failures and residency success [16].

This study was limited by the utilization of data from a single institution in an urban academic health center setting located in one region of the country. While it is limited in some aspects of applicability to residencies in different settings or with different resident characteristics, the findings of both positive correlations between cognitive exams and a lack of statistically significant, negative differences between residents with a history of academic challenges and their peers should be applicable to most Family Medicine residencies. This study can be used to inform further exploration of results across other primary care specialties and settings, particularly as it relates to academic challenges. The proportion of international medical graduates within the residency is representative of Family Medicine training nationally but may not be applicable to all institutions with significantly different ratios. Further, there were not enough residents within individual subsets of academic or professionalism problems to allow independent analysis of each. Additional exploration is needed across multiple institutions to allow further analysis of US allopathic cohorts, international medical graduate cohorts and predictive differences of application data points on residency readiness within the ACGME competency domains; this should include a mixture of urban academic and community-based settings from multiple regions of the country.

## Conclusion

We have found that incoming residents with a history of academic problems requiring medical school remediations, USMLE failures and professionalism concerns as a cohort perform similarly in residency to those without a history of these academic problems when evaluated across the six ACGME competency domains. We confirm, as is widely evidenced in the literature for other specialties, USMLE STEP 1 and STEP 2 CK are valid predictors of how residents will perform on the ABFM in-training exam as an intern; however, they do not predict resident preparedness across the six ACGME competency domains as evaluated in a clinical setting. Thus, rating and ranking of residency applicants with respect to the areas reviewed by this study support the continued concept of an ‘art in selection’ rather than a ‘science’ while providing data to support advocacy for applicants with a history of academic struggles.

## Data Availability

The datasets used and/or analyzed for this study are available from the corresponding author on reasonable request.

## Abbreviations

USMLE: United States Medical Licensing Exam
ACGME: Accreditation Council for Graduate Medical Education
ABFM: American Board of Family Medicine
NRMP: National Resident Match Program
UME: Undergraduate medical education
NBME: National Board of Medical Examiners
ERAS: Electronic Residency Application Service
MSPE: Medical Student Performance Evaluation
